# “We're Kind of in This Weird Space”: How Asian Americans physicians navigate perspectives towards race‐conscious admissions

**DOI:** 10.1111/medu.70247

**Published:** 2026-06-03

**Authors:** Candace J. Chow, Gavin Truong, Heeyoung Han, Tiffany Ho, Madeline Rogers, Zareen Zaidi

**Affiliations:** ^1^ Department of Internal Medicine & Director of Education Research, Office of Education Quality Improvement, Evaluation & Research University of Utah School of Medicine Salt Lake City UT USA; ^2^ Department of Internal Medicine The George Washington University School of Medicine and Health Sciences Washington DC USA; ^3^ Department of Medical Education & Director of Faculty Affairs in the Center for Human and Organizational Potential Southern Illinois University School of Medicine Springfield Illinois USA; ^4^ Division of Family and Community Medicine, Department of Family Medicine and Public Health University of Utah School of Medicine Salt Lake City UT USA; ^5^ Office of Education Quality Improvement, Evaluation & Research University of Utah School of Medicine Salt Lake City UT USA; ^6^ Division of General Internal Medicine, Co‐Director, Academy of Education Scholars, & Associate Director for Education Research & Scholarship The George Washington School of Medicine and Health Sciences Washington DC USA

## Abstract

**Introduction:**

(In)equity in admissions is a global conversation. Approaches to addressing these inequities include affirmative action, holistic admissions and race‐conscious admissions. Different countries vary in their usage of these approaches: while some have nationwide policies to implement affirmative action through quota systems, others utilise more holistic approaches, which are determined by individual institutions. This study explored the perspectives of Asian American physicians regarding race‐conscious admissions in medical education, using Poon and colleagues' multidimensional model of race class frames to understand how individuals navigate tensions between private and public interests.

**Methods:**

Using an interpretive qualitative approach, we employed an inductive‐deductive qualitative research methodology using purposeful, snowball sampling to identify participants. One‐hour interviews were conducted with 25 Asian American physicians across the Northeastern, Southern, Midwestern and Western regions of the United States. Interview transcriptions were analysed using inductive and deductive thematic analysis.

**Results:**

The majority of participants supported race‐conscious admissions, but both groups (supporters and non‐supporters) had fundamentally different interpretations of educational equity. We constructed five themes. (1) *Understandings of Race‐conscious Admissions* examines whether participants held accurate understandings of race‐conscious admissions policies; (2) *Intersectional Realities* versus *Individual Merit* explores how supportive participants viewed race and socioeconomic status as inextricably intertwined whereas unsupportive participants treated them as separate variables; (3) *Public* versus *Private Interests* demonstrates how supportive participants wanted to expand educational opportunities whereas unsupportive participants emphasised individual interests; (4) *Evolution of Consciousness* documents transformative experiences that facilitated movement from individualistic merit‐based frameworks toward systemic understanding; and (5) *Coalition* versus *Competition* illustrates divergent visions for Asian American physicians. Critically, widespread misunderstanding of race‐conscious admissions practices was identified, particularly among unsupportive participants.

**Discussion:**

This study challenges monolithic representations of Asian American perspectives on race‐conscious admissions, demonstrating significant heterogeneity that aligns with Poon's multidimensional framework. These results have significant implications for medical education policy, suggesting that efforts to build support for equitable admissions practices must address both factual misunderstandings and deeper philosophical differences about individual versus structural explanations for educational disparities. Future research should explore intervention effectiveness and examine how physicians' admissions policy attitudes relate to their clinical practice patterns with diverse patient populations.

## Abstract 2

This study shows that Asian American physicians largely support education equity and race‐conscious admissions, despite recent media suggesting otherwise. #MedEd

## 1 INTRODUCTION

Inequities in who is admitted to the medical profession exist globally.^1^, ^2^, ^3^, ^4^, ^5^ Different approaches to increasing equity in the admissions process, sometimes referred to as *widening access* (WA),^6^ have been utilised to address this problem, including affirmative action, holistic admissions and race‐conscious admissions. Nicholson & Cleland^6^ argue that much WA research lacks theoretical grounding, limiting its ability to address the root causes that render WA necessary. Applying theory to understand how medical professionals view WA allows us to examine these perspectives with nuance, through systemic factors. The focus of the present study utilises a raceclass framework^7^ to investigate how Asian American physicians perceive a particular approach to widening access: race‐conscious admissions.

### 1.1 Key concepts

Three key concepts are central to understanding diversity initiatives in medical education admissions. *Affirmative action* encompasses policies and practices within organizations seeking to include particular groups based on their gender, race, sexuality, creed or nationality in areas where they are underrepresented.^8^, ^9^ Affirmative action policies are often implemented as quota systems.^10^ *Holistic admissions* involves a comprehensive review of applicants that considers “experiences, perspectives, leadership, [and] accomplishments” rather than relying exclusively on standardised metrics.^7^ *Race‐conscious admissions* incorporates race and/or ethnicity as one factor within holistic admissions decisions to promote diversity, without establishing quotas or preferential treatment based solely on racial identity.^7^ Understanding these distinctions is crucial, as misconceptions that race‐conscious admissions promote quotas or grant automatic preference to applicants from certain racial backgrounds persist.^11^

The use of WA practices differs by country. Quota‐based affirmative action policies have been implemented in numerous countries, including the United States (US), Brazil,^4^ Sri Lanka,^2^ India^2^ and China,^12^ albeit in different ways. Although affirmative action in the US and Brazil has been used to impose quotas based on race and ethnicity,^1^, ^4^, ^13^ in Sri Lanka and India,^2^ quotas have been used to ensure that students from underrepresented castes and races are admitted by setting lower standardised entrance exam score thresholds for admission. In China, a quota system is used to ensure that admitted students represent diverse regions throughout the country by setting different test score thresholds by region.^12^ Schools in South Africa and the United Kingdom use more holistic approaches that include rurality and socioeconomic (SES) status.^1^, ^14^, ^15^

### 1.2 Widening access in the United States

For decades, US medical schools employed affirmative action and race‐conscious admissions policies to enhance physician workforce diversity. However, the 2023 Supreme Court ruling in “Students for Fair Admissions v. Harvard” has precipitated a fundamental reassessment of racial equity within medical education.^16^, ^17^, ^18^ This decision, which deemed the consideration of race unconstitutional, has been further interpreted by federal administrators as prohibiting any consideration of race in educational settings.^19^ Consequently, many medical schools and hospitals, concerned about potential loss of federal funding, have scaled back or eliminated diversity, equity and inclusion initiatives.^19^ Understanding whether faculty and administrators in the US will continue supporting equity initiatives in this transformed legal landscape is paramount to continue addressing persistent racial disparities in the US physician population.

These policies trace back to the civil rights era, when US President Kennedy's 1961 executive order first introduced the term *affirmative action*, followed by President Lyndon B. Johnson's 1965 executive order mandating its use in employment to prevent racial discrimination.^9^ After the Supreme Court prohibited the use of quotas in higher education, institutions maintained the ability to consider race in admissions, leading to race‐conscious and holistic admissions approaches designed to increase diversity.^13^ However, the 2023 Supreme Court decision eliminated race as a holistic admissions factor, requiring medical schools to identify alternative pathways to promote racial diversity and address race‐based educational inequities.^19^

### 1.3 The role of Asian Americans in WA conversations

The perspectives of Asian American physicians warrant particular attention in this evolving landscape, given their complex and nuanced relationship with educational equity policies.^7^ Although Asian Americans have historically supported affirmative action initiatives,^20^, ^21^ they are frequently mischaracterised as a homogeneous group uniformly opposed to such policies.^22^ This oversimplification fails to acknowledge the diversity of opinions within the Asian American community and neglects the complex historical and social contexts that shape their relationship with equity policies.^13^, ^23^

Asian Americans, defined as individuals with origins in East Asia, Southeast Asia or the Indian subcontinent,^24^ constitute a significant portion of the physician workforce. Although representing 7% of the general U.S. population, Asian Americans comprise 17.1% of active physicians, compared to 56.2% White, 5.8% Hispanic and 5.0% Black or African American physicians.^24^ This overrepresentation relative to their population has led to their classification as a “not under‐represented” minority group, distinguishing them from other racially minoritised groups in medicine.^23^

However, this classification obscures important complexities within Asian American communities. Historically, Asian Americans have occupied a liminal position in American racial hierarchies situated as better‐than Black and Brown Americans but less‐than White Americans.^25^ The *model minority* stereotype, which portrays Asian Americans as having achieved success surpassing other minority groups,^26^ was constructed to maintain white supremacy by creating divisions between Asian Americans and other communities of colour.^27^ This characterisation masks significant disparities in educational attainment across Asian ethnic groups and income levels,^28^ and overlooks the fact that certain Asian ethnic groups, including Filipinos, Vietnamese, Laotians, Cambodians and Hmong populations, remain underrepresented in the physician workforce.^23^ Despite its problematic origins, some Asian Americans have internalised the model minority stereotype, sometimes using it to position themselves above other communities of colour.^26^

Contemporary debates surrounding race‐conscious admissions in the US must be understood within this broader context of racialization and historical positioning. Although affirmative action policies were originally designed to address historic inequities faced by people of colour,^9^ opposition to these policies has intensified through recent litigation and executive actions, revealing considerable variability in public opinion on educational equity initiatives.^29^ Despite Asian Americans' substantial representation in the health care workforce and their potential influence in either supporting or challenging racial equity in medical education, limited research has examined their perspectives on affirmative action and educational equity policies.

Using Poon's multidimensional model of raceclass frames as our theoretical framework,^7^ our study explores Asian American physicians' views on educational equity in medical education. Our specific research questions are: (1) What are the perspectives and understandings of Asian American physicians concerning the impact of racial/ethnic identity and socioeconomic status on educational achievement? (2) How do the lived experiences of Asian American physicians shape their views about policies designed to create educational equity, specifically race‐conscious admissions? (3) How do Asian American physicians believe educational equity should be addressed in medical education moving forward?

## 2 THEORETICAL FRAMEWORK

This study employs Poon et al's^7^ multidimensional model to understand how Asian Americans’ views on race and class shape their attitudes toward educational equity and race‐conscious college admissions. The model is portrayed as a quadrant and with views of race and class (either as interconnected or separate factors) along the x‐axis and views of education (as private vs. public interests) along the y‐axis. The model posits that systemically connected views of race and class are what predict individuals’ support of race‐conscious admissions. We chose Poon's model because it provides a critical and systemic lens for understanding the affirmative action debate by highlighting how understandings of race and class work together to inform individual perspectives.

The framework identifies four distinct viewpoints (one in each quadrant):

*Ethnocentric Nationalism*: Views education as a private benefit and takes a colour‐blind approach to racism and economic inequality. Attributes educational disparities to cultural differences or individual traits rather than systemic barriers, often viewing one group's values as superior to others.
*Abstract Liberalism*: Sees education as a public good but explains inequities primarily through socioeconomic rather than racial factors. Emphasises individual merit and equal opportunity, believing success is achievable for anyone given equal access to resources.
*Conscious Compromise*: Treats education as a private interest but recognises systemic racism and economic inequality. Supports race‐conscious admissions as a pragmatic strategy to promote equity, even without full ideological conviction.
*Systemic Transformation*: Views education as a public interest requiring structural change. Sees race‐conscious admissions as essential for addressing historical racism and increasing access for underrepresented groups.

We applied this framework throughout our research process in developing the research questions, designing interview questions and analysing data to explore how participants’ race and class perspectives influenced their views on admissions policies.

## 3 METHODS

### 3.1 Research approach

This study adopts an interpretive qualitative approach to explore how participants make meaning of their experiences within medical education. In‐depth, semi‐structured interviews elicited rich, contextual descriptions of participants’ experiences and perceptions. Following principles of qualitative analysis,^30^ a combination of inductive coding and deductive thematic analysis was employed to identify patterns of meaning across participants’ accounts. We paid specific attention to participants’ interpretations of race‐conscious admissions and how those beliefs were shaped by life experiences, personal identity and professional socialisation.^31^

### 3.2 Study Design and Recruitment

We used the concept of information power to guide our decisions about sample size.^32^ Information power provides criteria by which to gauge the richness of the information provided by participants and decide when to cease data collection. We anticipated approximately 20–25 interviews in our sample based on a narrow study aim, sample specificity and use of a guiding framework.^7^ Attending physicians were recruited using purposive, voluntary, stratified and snowball sampling.^33^ We focused on attending physicians because they are best positioned to engage in conversations regarding educational equity and admissions policies. Although some admissions committees include students and trainees, not all do. We aimed for representation across a range of sociocultural and professional dimensions, including geographic region, ethnic background (East, Southeast, South Asian), generation status (e.g. immigrant vs. US‐born), gender identity and practice setting (academic vs. community‐based, primary vs. specialty care). We did not actively recruit participants from the Middle East, as they are not usually classified as Asian. Recruitment began with outreach to academic and professional networks. Interested individuals completed a brief screening survey based on Bullock et al's^34^ survey to support sample stratification that included questions about demographic background and whether individuals were supportive of educational equity and race‐conscious admissions. Additional participants were identified through snowball referrals.

### 3.3 Data Collection

Participants received e‐mail invitations explaining the study's purpose and participants' potential contributions to knowledge. We reinforced that participation was voluntary. A total of 30 individuals completed the screening survey. One participant who initially agreed to be interviewed later declined. Four survey respondents did not respond to invitations for interviews, which yielded a sample of 25 participants. A semi‐structured interview guide was developed based on the research team's lived experience, existing literature on Asian Americans' experiences in medicine, and existing literature related to race‐conscious admissions (Appendix 1). The interview guide was pilot‐tested and refined. Interviews explored participants' background, reflections on personal identities and lived experiences, as well as their beliefs about educational equity and race‐conscious admissions.

Three team members (CJC, TH, HH), all experienced in qualitative interviewing, conducted the interviews. After piloting the questions with each other, we used the guide to conduct semi‐structured, one‐hour interviews via Zoom between July 2024 and April 2025. The interviewers met during the interview process to discuss participant responses. These peer debriefing sessions served as opportunities to reflect on our own positionalities in relation to the participants' and what they discussed. All interviews were audio‐recorded and professionally transcribed. Transcripts were de‐identified to protect participant confidentiality and uploaded into Dedoose (v10.0) for collaborative analysis.

### 3.4 Data Analysis

We analysed data using an inductive‐deductive thematic analysis approach,^30^ identifying themes from the data set while also being informed by our existing theoretical framework of *raceclass frames*.^7^ We approached the transcripts inductively, allowing participant narratives to guide the generation of open codes. As a team, we jointly coded three transcripts over the course of two meetings to align our interpretations and to construct a preliminary codebook. We then independently reviewed a subset of transcripts and met monthly to discuss interpretations, refine codes and collaboratively construct a shared codebook. During this process, our coding did not diverge substantially; for example, coding of participants' interactions within Asian American/racially minoritised communities was similar. After reaching consensus on code definitions, each transcript was coded by two team members. Discrepancies were resolved through group discussions. Although there were no major discrepancies, we wrestled with describing some concepts with ample nuance, such as how participants conceptualised the ideas of *giving back*, *allyship* and the *model minority myth*. All codes were eventually refined deductively using our theoretical framework. The analysis was conducted iteratively with the group adjusting themes based on theoretical insights.

We also categorised participants' position on race‐conscious admissions as supportive or unsupportive and assessed the accuracy of their understanding on this topic. To determine support, we used participants' self‐reported opinions, explicitly asked on the screening survey and in the interview. To determine the accuracy of understanding, participants were asked during interviews to elaborate on what it meant and why they supported/did not support this approach. Participants who described race‐conscious admissions as involving a holistic process where race was just one factor were categorised as having an accurate understanding. Participants who described race‐conscious admissions as reaching a quota, that it resulted in an “Asian American penalty” ^13(p. 57)^, or that while it could help in principle but was unfair in practice were categorised as having an inaccurate understanding. Finally, though participants reported how they felt about educational equity and race‐conscious admissions on the screening survey, we used interviews to confirm these positions and ask participants for elaboration.

### 3.5 Trustworthiness and author positionality

Our research team consisted of five Asian American scholars (one Vietnamese American, one Korean American, two Chinese Americans and one Iranian‐born Pakistani American) and a White American scholar. We bring a range of generational perspectives, from first‐generation immigrants to multigenerational Americans, as well as diverse professional roles across academic medicine and medical education including practicing physicians, physician educators and health professions education researchers and research assistants.

Our shared racial and professional identities allowed us to build rapport with participants and attune to cultural and experiential nuances throughout the research process. We also acknowledged potential for over‐identification and the influence of our own positionality as Asian American professionals within elite educational and health care systems on the stories our participants chose to share with us. Our shared position in supporting race‐conscious admissions was another commonality that we acknowledged as we interviewed participants who shared different perspectives. We engaged in regular reflexive practices, including peer debriefings, to interrogate our assumptions and ensure that we were preserving the complexity of the narratives shared with us.

### 3.6 Ethics approval

This study was deemed exempt by the University of Utah Institutional Review Board (IRB_00176202). Data sharing agreements were created with The George Washington University and Southern Illinois University to allow all members of the research team to engage in analysis.

## 4 RESULTS

### 4.1 Summary of demographic characteristics

A total of 25 Asian American physicians participated (Table 1). They represent all four US census regions and practice in diverse clinical and institutional settings. Participants brought a range of perspectives informed by generational status and lived experience with race and equity in education and medicine. Again, support was self‐reported by participants. All but one participant believed that students should have an equal opportunity to experience high‐quality education. Most participants in this study were supportive of race‐conscious admissions: 19 participants reported support for race‐conscious admissions, four said they were unsupportive and two reported being neutral. With regard to region, more participants were supportive than unsupportive in each region. There were more women (N = 18) participants in the study than participants of other genders, and most participants in each gender were supportive. With regard to generational status, most participants were the child of immigrants (N = 17) and only those in this generational category identified as unsupportive. Of the 11 participants who had admissions experience, eight were supportive. Eight of the participants who identified as Chinese were supportive, as well as those who identified as Vietnamese and South Asian. Importantly, individuals who were supportive vs. unsupportive of race‐conscious admissions did *not* differ with regard to a single demographic characteristic (i.e. not all women participants felt the same way). Instead, participants of varying ethnicities, regions and generational statuses spanned both groups, illustrating that perspectives are informed by multiple factors.

**TABLE 1:** Participant demographics (note that some participants identified with multiple sub‐categories).

Total number of participants	25
Supportive of educational equity	24
Supportive of race‐conscious admissions	19
US Census region	
*Northeast*	6
*South*	6
*Midwest*	3
*West*	10
Type of practice	
*Academic*	15
*Community clinic/hospital*	9
*Another setting*	4
Urbanicity	
*Urban*	19
*Suburban*	9
*Rural*	2
Generational status	
*Immigrant*	7
*Child of immigrants*	17
*Child of parents born in US*	1
Gender	
*Woman*	17
*Man*	6
*Non‐binary*	1
*Genderqueer*	1
Ethnicity	
*Vietnamese*	3
*Indian*	4
*Chinese*	13
*Taiwanese*	3
*Korean*	1
*Pakistani*	1
*Multiple ethnicities*	2

### 4.2 Organisation of themes

Theme 1 examines whether participants held accurate understandings of race‐conscious admissions policies, establishing a foundational knowledge baseline before exploring their experiential perspectives. Themes 2 through 5 were organised using Poon's framework as an analytical lens, mapping participants' narratives onto the model's quadrants to illuminate how Asian American physicians conceptualise lived experience, educational equity and race‐conscious admissions.

Theme 2 (*Intersectional Realities* versus *Individual Merit*) captures how participants analysed race and SES as intersecting structural forces or discrete variables and how these analytical orientations informed their positions on race‐conscious admissions, corresponding to the left–right axis of Poon's model. Theme 3 (*Public* versus *Private Interests*) examines how participants' views of education as a public or private good shaped their stances on equity‐focused policy, mapping onto the model's vertical axis. Together, Themes 2 and 3 address research questions 1 and 2, exploring how racial/ethnic identity, SES and lived experience shape Asian American physicians' views on race‐conscious admissions. Theme 4 (*Evolution of Consciousness*) further addresses these questions by tracing how participants' perspectives shifted over time, with some describing movement from right‐ to left‐quadrant orientations in Poon's framework. Theme 5 (*Coalition* versus *Competition*) addresses research question 3, examining participants' visions for how educational equity should be pursued in medical education and reflecting their broader ideological alignment within the model.

Research questions one and two [(Q1) What are the perspectives and understandings of Asian American physicians concerning the impact of racial/ethnic identity and SES on educational achievement? (Q2) How do the lived experiences of Asian American physicians shape their views about policies designed to create educational equity, specifically race‐conscious admissions?] are addressed in Themes 2, 3 and 4. Research question three is addressed in Theme 5: (Q3) How do Asian American physicians believe educational equity should be addressed in medical education moving forward?

### 4.3 Theme 1: Understandings of Race‐Conscious Admissions

Sixteen participants held accurate understandings of race‐conscious admissions, describing it as using race as one factor in a holistic process. Participant 2 described it as: “taking someone's racial and ethnic background and really thinking about the opportunities that they might have or might not have had because of that into consideration … in making admissions decisions.” It is important to note that not everyone in any group (supportive, unsupportive, neutral) had an accurate understanding of race‐conscious admissions. In fact, a critical finding was the widespread misunderstanding of race‐conscious admissions practices, particularly among unsupportive participants. They described it as a quota and as something that harmed Asian American applicants, which stood in contrast to how supportive physicians described the process, with one even noting that race‐conscious admissions is “not a zero‐sum game” (P2). Unsupportive physicians believed race‐conscious admissions was designed to achieve “equity of outcome,” meaning “the final class picture at this university medical school has to look like XYZ” (P20). Participant 5, despite having admissions experience, incorrectly described the process: “You look at their standardized test scores and X, Y, Z, assign numbers to them … then sometimes their race gives them additional points or some sort of advantage just because they come from a certain race.” Importantly, all participants with admissions experience who were unsupportive or neutral held incorrect definitions, whereas one supportive participant with such experience held an inaccurate understanding.

### 4.4 Theme 2: Intersectional Realities versus Class‐Based Merit ‐ Competing Understandings of Educational Opportunity

Our analysis revealed fundamental differences in how participants conceptualised the relationship between race, SES and educational opportunity. Participants supporting race‐conscious admissions demonstrated what Poon categorises as *systemic transformation* or *conscious compromise* thinking. A total of 18 participants viewed race and SES as inextricably intertwined structural forces, aligning with either the systemic transformation or conscious compromise viewpoints from Poon et al's framework. These physicians said, “we know in the US both race and income and socioeconomic status really constrain the opportunities that you have” (P2), explaining educational inequities as products of intersecting systems of disadvantage. They believed “race should totally be part of [the] application” (P21) and supported holistic admissions that included race as a factor because they believed that racial identity fundamentally shaped lived experience and perspective.

In contrast, participants opposing race‐conscious admissions reflected *abstract liberalism* and *ethnocentric nationalism* frames (N = 7). For the most part, these participants treated race and socioeconomic status as separate variables. These physicians argued for colour‐blind meritocracy, expressing concerns that Asian applicants are regarded more negatively: “might have a very Asian‐sounding last name…and then people can figure it out…Because once you're at that stage of late career or even in college, I really think it should just be about your merit” (P12). They conceptualised socioeconomic factors as more legitimate considerations: “ … I think [SES] are just as correlated to outcomes…without having to bring in race” (P5). The ethnocentric nationalism perspective was reflected by participants who attributed educational disparities to racial or cultural deficiencies rather than structural inequities. Participant 15's observed: “that mostly Black community in America. I always worry about them. I said, ‘Why they're not able to move forward?’…We always think … that education is the fundamental … Or sometimes if they have barriers they need to be helped more … Hopefully then they will assimilate and they will advance their cause …” Although this participant is sympathetic to the poverty experienced by many Black patients he has worked with and believes they must be given resources to change their situation, the root of the cause, in his mind, is due in part to individual causes, including a lack of “assimilation.” Interestingly, this participant identified as being *supportive* of race‐conscious admissions but held inaccurate understandings of what it was (i.e. that it should only be implemented on a temporary basis so that those given resources would not become “entitled.”) Participant 20 remarked, “I think my parents brought over culturally this desire of hard work and accomplishment and all this … my parents came over doing some of the toughest, low skill jobs … And in one generation their kid grew up to be a military officer and a highly trained [specialty] physician in this country. That's incredible ….” This framing positions Asian immigrant success as evidence that systemic barriers are surmountable through individual, fbootstrapping effort.

Critically, *both* groups acknowledged that race and SES impact educational opportunities. One unsupportive participant noted, “there are cultural and historical things that have happened that some segments of the population are at more disadvantaged than others” (P5). Yet, despite the universal acknowledgement that inequities have structural histories, not all participants viewed current factors this way. Although supportive participants viewed differential outcomes as structural: “even though you can grow up in [the same place] and have similar kind of backgrounds, you may not always end up in the same spot later on in life, even though you really want to” (P3), unsupportive participants questioned current individual motivation as the main contributor to inequities: “What if XYZ group wasn't as interested in being in that group?” (P20).

### 4.5 Theme 3: Public vs. Private Interests ‐ How Personal Narratives Shape Policy Perspectives

Participants' contextual experiences fundamentally influenced their positioning within Poon's framework, particularly regarding the tension between private interests and the public good. Participants in both groups shared similar backgrounds of parental sacrifice and varying degrees of privilege, yet interpreted these experiences through markedly different analytical lenses.

#### 4.5.1 Transforming Sacrifice and Privilege into Public Service

Physicians supporting race‐conscious admissions who embodied systemic transformation orientations reflected on how personal experiences of sacrifice and privilege motivated them to expand opportunities for others. These participants viewed their success as contingent on pivotal moments of institutional support. Participant 1, who “grew up waiting tables and doing homework at the restaurant,” recognised how their mother's tuition reimbursement benefits “open[ed] the doors for me to move forward in my career… Had [my parent] not been able to do that, I honestly don't know if I'd been able to be where I am professionally.” This recognition fostered a “passing it on” (P24) mentality, where professional success created obligations to support others who were structurally disadvantaged. Participant 7 exemplified this approach: “I purposely bought a whole year's worth of [prep questions]… even though I didn't need it, [thinking] ‘Okay. Now that I have this, I'm going to pass my login and whatever to the residents that… needed it and couldn't afford it…”

Supportive participants also rejected individual meritocracy, instead defining the “best” applicants through a conscious compromise lens that prioritised diversity's benefits: participants spoke about how race‐conscious admissions was one way to “have more Black and Brown students enrolled and supported in [medicine]” to achieve racial concordance between physicians and patients. They demonstrated comfort with uncertainty regarding their own children's educational outcomes, trusting that “the rest of it will life take on its own track” (P23).

#### 4.5.2 Individual and Private Interests

Unsupportive participants were less interested in “passing it on,” at least to everyone, because they recounted experiences where they felt they had been wronged because of their race. Their personal experiences of perceived admissions rejections were interpreted through frameworks of individual competition rather than structural analysis. Participant 4 recalled: “ I thought that my scores were high enough to get into a Johns Hopkins or Cornell or UPenn, or maybe I was just delusional. I don't know. But I knew other people with different stats that got in…. but I do think that maybe the race had something to do with it.”

Significantly, several unsupportive participants reported workplace experiences of being marginalised within diversity initiatives, creating tension between their own experiences of exclusion and their resistance to race‐conscious policies. Participant 19 shared: “In [location] it was Black diversity was basically the big one, and they did a really good job…I have nothing against it, I think it's amazing, but I did make a comment to her … I was like, ‘Well, I'm the only Asian person here. We need more.’… And she's like, ‘That's not the diversity we're looking for.’ And I was like, ‘Well, that's rude.’”.

Importantly, unsupportive participants also recognised how fortunate they were, explaining “oh wow, I'm able to afford this or to provide this for my family” (P6) and acknowledging that not all families can do the same. Even so, they remained anchored in abstract liberalism thinking that prioritised individual merit and private interests (outcomes that benefitted themselves, their loved ones, and those they believed were deserving). This group's abstract liberalism extended to solutions for educational inequity, emphasising individual responsibility: “I would argue if the circumstances for that particular institution are not ideal, then the onus is on you or your family to try to improve that situation or you make the best of the situation that you have” (P4). Participant 15 expressed concern that extended assistance created dependency: “it ingrained in people's mindset that this becomes rights…then it becomes entitlement.” The tension between public good and private interests was particularly acute regarding their own children. Participant 6 admitted: “I think from a personal, selfish standpoint, that I struggle with, ‘Well, if it's going to put my family or my children at a little bit of a disadvantage, then my brain says, ‘I don't want to do that either.’“.

Finally, they also viewed medical competency through technical lenses, arguing that cultural humility or racial concordance with patients should not be deciding factors because they were not as important as “objective” (P4) test scores or “merit” (P12). P20 explained, “If I'm under anesthesia and someone's cutting me open, I want to know that they were super, super smart, super hard‐working, they studied all the time. They were some of the best of the best” (P20). Unsupportive participants interpreted incompetence through a merit‐based lens, leading them to believe that racialised minority status was to account for clinical incompetence.

### 4.6 Theme 4: Fluidity of Consciousness – How Critical Consciousness Shapes Perspective Transformation

A remarkable finding was the documented evolution from abstract liberalism/ethnocentric nationalism to systemic transformation thinking among several supportive participants (N = 9), suggesting that exposure to diverse experiences can fundamentally shift analytical lenses within Poon's model and influence critical consciousness. None of the participants spoke about changing views in the other direction (systemic transformation/conscious compromise to abstract liberalism/ethnocentric nationalism).

#### 4.6.1 Patient Care as Catalyst for Understanding

Participants described transformative patient encounters that challenged their initial meritocratic assumptions. Participant 3 reflected:

“if I'm totally honest, probably in my twenties…I think I would've just [said] ‘Everyone should just work really hard… But now that I'm older, and I've seen different scenarios…I recognize that isn't the case for everyone. And even though you can grow up in [the same place] and have similar kind of backgrounds, you may not always end up in the same spot later on in life, even though you really want to.”

This participant's journey illustrates movement from individual‐focused thinking to recognising structural constraints as “not a choice” (P8).

#### 4.6.2 Educational Experiences as Transformative Moments

Educational opportunities, particularly exposure to Asian American identity development programmes, proved catalytic for perspective shifts. Participant 10 described a pivotal undergraduate experience:

“I remember sitting in a conference room as a freshman in college. It was one of those gathering conferences, one that was Southeast Asian organizations based…I came away, I was like, ‘Oh, that's interesting.’ And it started by the practice of re‐evaluating everything that I had accepted as unquestionable truth from how my parents raised me.”

Exposure to diverse classmates also shifted thinking: participants reported that interactions with peers made them realize “that some people do need more assistance, and that is equal to equity” (P14).

#### 4.6.3 Professional Observations Challenging Meritocracy

Professional observations of privilege in action proved particularly transformative in revealing how structural advantages masquerade as individual achievement. Participant 22 described her awakening: “at some point in my life I definitely was like, ‘Oh no, only the best should get [in]. It should be based on how good you are.’ Then probably post‐college is when I realized, all the white guys got really great finance jobs, and… it was so easy for them. It was so freaking easy.”

Many participants could not identify specific “aha moments” (P13) but described gradual evolution “over time… after just reading things” (P13), suggesting that sustained exposure to alternative frameworks can facilitate the development of critical consciousness and movement between Poon's typologies.

### 4.7 Theme 5: Coalition versus Competition ‐ Divergent Visions for Asian American Professional Identity

Both groups recognised the need to address “significant underrepresentation of Asian Americans…in academic medicine and…leadership in medicine” (P16), yet proposed fundamentally different strategies reflecting their underlying frameworks for understanding equity, merit and professional responsibility in medical education. Participants' perspectives on race‐conscious admissions reflected broader philosophical differences about Asian American physicians' professional responsibilities and community allegiances, mapping onto Poon's framework regarding private versus public interests.

#### 4.7.1 Systemic Transformation and Coalition Building

Supportive participants embraced systemic transformation approaches, advocating for coalition‐building across communities of colour despite acknowledging Asian Americans' liminal position. As Participant 17 noted: “We're kind of in this weird space of we're sometimes seen as people of colour, sometimes not seen as people of colour, of where we're seen as overrepresented in medicine.” These physicians sought to “speak out in favour of educational equity” (P2) while simultaneously addressing underrepresentation among Asian ethnic subgroups. They also spoke about recognising the racial privilege of being Asian American while also experiencing racial minoritisation. Their vision of systemic transformation extended beyond admissions to ongoing support structures. Participant 2 emphasised: “I think another area in both undergrad and graduate medical education where I don't think we do enough is supporting folks once they're actually here… we admit a diverse class, box checked…but it's also…making sure that once you're here…we feel confident that we can elevate and support you to and we don't contribute to perpetuating bias.”

#### 4.7.2 Abstract Liberalism and Merit‐Based Advocacy

Unsupportive participants, reflecting abstract liberalism orientations, advocated for “dissolution of trying to make a program equitable [with respect to outcomes], but rather more so on merit” (P4). Although supporting mentorship and professional development for students “that are less prepared” (P18), they disconnected these activities from racial or ethnic identity, preferring ostensibly colour‐blind approaches to professional service.

## 5 DISCUSSION

Our study reveals the heterogeneous perspectives among Asian American physicians regarding race‐conscious admissions in medical education, challenging monolithic representations that emerged during the 2023 Supreme Court proceedings. Our findings demonstrate that Asian American physicians' views align with Poon and colleagues' multidimensional model of race‐class frames,^7^ with participants embodying all four typologies: ethnocentric nationalism, abstract liberalism, conscious compromise and systemic transformation. Additionally, our research points to ongoing debates about meritocracy and its role in admissions, how misunderstandings of holistic admissions affect perspectives, how physician perspectives can change over time, and the ongoing liminal roles of Asian Americans in these conversations.

Our analysis shows that while some supportive and unsupportive participants share similar demographic backgrounds and acknowledge that race and socioeconomic status impact educational opportunities, no one demographic factor accounted for differences in perspectives. Instead, participants fundamentally differed in their interpretation of these impacts and their proposed solutions. Supportive participants viewed disparities as products of intersecting structural forces requiring systemic interventions, whereas unsupportive participants attributed differences to individual choices and cultural factors, advocating for colour‐blind meritocratic approaches. These findings differ slightly from those in Poon et al's^7^ work, which found that opponents of affirmative action were mainly Chinese immigrants. Nonetheless, our findings align with and extend existing scholarship on Asian American political attitudes and educational policy preferences.^7^, ^35^ The diversity of perspectives we documented supports research demonstrating that Asian Americans do not constitute a monolithic voting bloc, with significant variation in policy preferences across ethnic subgroups, generational status and socioeconomic backgrounds.^36^, ^37^

Interestingly, unsupportive physicians, many of whom were immigrants themselves or the children of immigrants and who have benefitted from the social mobility that practicing medicine affords, were resistant to race‐conscious admissions because they believed it was antithetical to meritocracy. They preferred a system that rewarded equal opportunity and merit. And yet, rather than producing equal opportunity, meritocracies reproduce hierarchy.^38^

This tension we identified between individual merit and structural transformation is reminiscent of ongoing debates regarding admissions in higher education.^38^ Specific to medical education, these findings also point to broader debates about competency‐based versus equity‐focused approaches to training and assessment.^39^ Our findings reveal how physicians' understandings of structural competency and health equity may influence their perspectives on admissions policies.^16^, ^40^, ^41^ Thus, participants who were unsupportive of race‐conscious admissions might be presumed to oppose such policies in principle.

Consistent with Poon and colleagues,^7^ we identified widespread misunderstandings of race‐conscious admissions practices, particularly among unsupportive participants. Individuals who do not support race‐conscious admissions do not do so simply out of principle. They also seem to believe race‐conscious admissions are problematic in practice, with all four unsupportive physicians who had admissions experience holding incorrect definitions of how these policies function. The documented misunderstandings of race‐conscious admissions echo broader research on policy misperception and its impact on public opinion,^13^ suggesting that accurate information about how holistic admissions actually functions could influence attitudes.^42^, ^43^

The study illuminates how personal experiences including patient care encounters, educational opportunities and professional observations can facilitate evolution from individualistic merit‐based frameworks toward a systemic understanding of structural inequities. Our finding that personal experiences can facilitate movement between Poon's typologies contributes to literature on political socialization and attitude change, particularly the role of intergroup contact and professional experiences in reshaping racial attitudes.^44^, ^45^ This finding also validates literature on how critical consciousness is developed. Critical consciousness, or *conscientization*, was conceptualised by Brazilian educator Paolo Freire to describe the process of understanding one's own experiences and social positionality in reference to others' experiences and positionalities.^46^ Medical education scholars have posited that physicians must develop critical consciousness to provide culturally relevant care because one needs to understand oneself in relation to their patient to provide the best patient care possible.^47^, ^48^ Participants' development of critical consciousness through patient encounters and professional experiences suggests that this process in medicine is cyclical, whereby exposure to individuals and experiences different from one's own can catalyse critical consciousness, which can then change perspective.

Harkening back to the “weird space” invoked in this paper's title, our findings illuminate the liminal position Asian American physicians occupy within diversity frameworks ‐ sometimes recognised as people of colour, sometimes not ‐ a positioning that aligns with scholarship on model minority mythology and its documented impact on interracial coalition building in health care settings.^49^ This liminality is not merely symbolic; it shapes how participants navigated, rationalised, and at times resisted dominant narratives about race‐conscious admissions. The finding that supportive physicians aspire toward cross‐cultural coalition reflects a broader conviction that equity cannot be achieved in isolation. This stance converges with evidence that workforce diversity in health care yields measurable benefits for all patients and with the structural argument, raised by many participants themselves, that quality health care is inseparable from the active dismantling of health inequities at the systems level.^50^, ^51^

Overall, our application of Poon's framework provides empirical validation of the model's utility in understanding how individuals navigate the tension between private interests and public good in educational policy contexts.^7^, ^52^ One point that surfaced during our study that was not addressed by Poon et al's was specific to medical school: the gravity of training physicians, who take care of people's health, and the stakes that raises for deciding who is qualified to be admitted.

### 5.1 Implications for research and practice

These findings have significant implications for medical education policy, faculty development and future research directions. First, our finding that the widespread misunderstanding of race‐conscious admissions practices among physicians suggests an urgent need for faculty development programmes that clarify how holistic admissions actually function, moving beyond misconceptions about quotas or point systems. Medical schools should consider implementing educational initiatives that help admissions committee members and the broader academic medicine community understand the nuanced ways race intersects with other aspects of applicant experience and perspective. Such initiatives could include fact‐checking and debunking of incorrect information, which are techniques that have proven at least somewhat effective in changing beliefs about vaccines.^53^, ^54^, ^55^ For example, schools could create an interactive workshop that details what race‐conscious admissions is believed to be and what it actually is. They could share the history and definitions of affirmative action, holistic admissions and race‐conscious admissions and explain what selection would look like under each of these approaches to contrast and show how race‐conscious admissions is very different from meeting a quota under affirmative action.

Second, our documentation of perspective evolution suggests that targeted interventions including structured intergroup contact, patient care experiences with diverse populations, and education about structural determinants of health may facilitate movement toward more systemically informed viewpoints. The finding that professional observations of privilege can be particularly transformative indicates that creating opportunities for physicians to witness and reflect on structural advantages may be valuable for developing equity and critical consciousness. Researchers have demonstrated effectiveness in raising medical learners' critical consciousness through a variety of pedagogical approaches: guided discussion,^56^, ^57^ explicit exposure to issues of privilege and inequity,^57^ and even theatrical exercises.^58^ The faculty development initiatives mentioned above might extend beyond fact‐checking and debunking and could make use of targeted discussions and reflections on their own interactions with diverse patient populations to encourage critical consciousness.

Third, the study highlights the need for more nuanced approaches to engaging Asian American physicians in diversity, equity and inclusion efforts, recognizing both their potential contributions to coalition‐building and their experiences of marginalization within existing frameworks. The history of Asian American political involvement is strong.^59^, ^60^ Teaching this history and the legacy of Asian American political activism, both among Asian groups and across communities of colour, could be one way to spur more interracial coalitions.

Future research should explore the effectiveness of different educational interventions in shifting perspectives on race‐conscious policies, including whether addressing misinformation through fact‐checking and debunking is effective, examine how Asian American physicians' views influence their mentorship and advocacy behaviours, and investigate the relationship between physicians' admissions policy attitudes and their clinical practice patterns with diverse patient populations. Future work could also examine this internationally to understand how Asians in different countries navigate their own admissions policies. Additionally, longitudinal research could track how perspectives evolve over career trajectories and identify critical periods for intervention. Our study shows how physician views on racial/ethnic identity and socioeconomic status influence perspectives of educational achievement. Future studies could further study whether differences in views exist by Asian ethnic groups. From a policy standpoint, these findings suggest that efforts to build support for equitable admissions practices must address both factual misunderstandings and deeper philosophical differences about individual versus structural explanations for educational disparities.

### 5.2 Limitations

One of the limitations of our study was the small population size. We limited participants to practicing physicians, as we wanted to gain insights from their lived experiences practicing as health care providers, as academics, and through their personal experiences related to affirmative action and race‐conscious admissions with friends and family. Many of our participants live and practice in the Western United States in areas where progressive beliefs abound, which may be a confounding factor in influencing participant perspectives and only reflect perspectives within a US context. Additionally, as with most qualitative interview studies, participants were asked to reflect on life experiences which introduces potential recall bias. Including representation from medical students and trainees may have increased our sample size and yielded additional perspectives. Second, because of the small sample size, we were unable to comment specifically on Asian ethnic subgroups and how perspectives vary across these subgroups. Although we tried to ensure casting a wide recruitment net, it is possible that physicians who were more willing to discuss sensitive topics agreed to take part in the study. Despite the limitations, the rich data set provides information for other researchers to build on our work.

## 6 CONCLUSION

Our study challenges the prevailing narrative that Asian Americans uniformly oppose affirmative action by highlighting the diversity of perspectives among Asian American physicians. Most participants in our study supported race‐conscious admissions in the medical school selection process. They were thoughtful about their approach to the topic and understood the need to focus on uprooting structural racism and working towards systemic transformation for equity in medical education. They further understood racism's effects on people and the ways that economic background and life experiences interconnect. Our study lays the foundation for subsequent work on this critically important topic by introducing nuance to a greater conversation on educational equity.

## AUTHOR CONTRIBUTIONS

**Candace J. Chow:** Conceptualization; investigation; funding acquisition; writing—original draft; methodology; writing—review and editing; formal analysis; supervision. **Gavin Truong:** Writing—original draft; writing—review and editing; formal analysis. **Heeyoung Han:** Conceptualization; writing—review and editing; investigation; formal analysis; methodology. **Tiffany Ho:** Investigation; writing—review and editing; formal analysis. **Madeline Rogers:** Formal analysis; writing—original draft; writing—review and editing. **Zareen Zaidi:** Conceptualization; writing—review and editing; methodology; writing—original draft.

## USE OF AI

During the preparation of this work, the authors used Claude.ai to help make sentences succinct. After using this tool/service, the authors reviewed and edited the content as needed and take full responsibility for the content of the publication.

## Data Availability

The data that support the findings of this study are available on request from the corresponding author. The data are not publicly available due to privacy or ethical restrictions.

## APPENDIX 1 INTERVIEW QUESTIONS

- Probe: Identity Questions
  - Please tell me about yourself (background – family, work, etc.)
  - What identities are important to you and why? Please share specific examples of experiences that have influenced how you view yourself
    1. Sub‐probes: If the participant does not mention, then inquire about the following identities specifically:
      - How do you identify racially and ethnically?
      - How do you identify with respect to socioeconomic status?
      - What other identities are important to you?
      - What is your immigration history/family's immigration story?
      - How has your understanding of race/ethnicity changed over time/evolved, if at all?
- Probe: Educational Equity Questions
  - In the survey, you said X. How would you define educational equity and how did you arrive at this definition?
    1. Sub‐Probe:
      - Can you elaborate?
      - What (else) led to you thinking this way?
      - Please share examples of how you have experienced educational equity/inequity
      - Did you experience any sacrifice/struggles that have informed how you think about equity?
  - In the survey, you said Y. What does race‐conscious admissions mean to you?
    1. Subprobes:
      - Can you elaborate?
      - What led to thinking this way?
      - Do you think race‐conscious admissions is related to educational equity? Do you think you have benefitted from or been hurt by race‐conscious admissions?
      - Did you experience any sacrifice/struggles that have informed how you think about race‐conscious admissions?
    2. Sub‐Probe: Please share your personal story about your personal experiences related to undergraduate and graduate degree programs in relation to the application and the admissions process.
    3. Sub‐Probe: Have you had any experiences with children, extended family, or friends that have shaped your view of race‐conscious admissions?
  - The U.S. Supreme Court recently ended race‐conscious admission programs at colleges and universities across the country. What are your thoughts about the recent US Supreme Court decision regarding race‐conscious admissions?
- Probe: Addressing Educational Equity
  - Should the field of undergraduate medical education address educational equity in the admissions and selection processes? (Ask about GME, CME if there's time to ask)
  - If so, how?
  - What is the role that Asian/Asian American physicians should play in addressing educational equity?
- Closing
  - Please tell me other thoughts/comments/experiences that I did not ask for but that you wanted to share.
